# Low-dose calcium supplementation for preventing pre-eclampsia: a systematic review and commentary

**DOI:** 10.1111/1471-0528.12613

**Published:** 2014-03-13

**Authors:** GJ Hofmeyr, JM Belizán, P von Dadelszen

**Affiliations:** a Effective Care Research Unit, East London Hospital Complex/University of the Witwatersrand/University of Fort HareEast London, Eastern Cape, South Africa; b Institute for Clinical Effectiveness and Health Policy (IECS)Buenos Aires, Argentina; c Department of Obstetrics and Gynaecology and Child and Family Research Institute, University of British ColumbiaVancouver, BC, Canada

**Keywords:** Calcium replacement, calcium supplement, eclampsia, low-dose calcium, pre-eclampsia

## Abstract

**Background:**

Epidemiological data link low dietary calcium with pre-eclampsia. Current recommendations are for 1.5–2 g/day calcium supplementation for low-intake pregnant women, based on randomised controlled trials of ≥1 g/day calcium supplementation from 20 weeks of gestation. This is problematic logistically in low-resource settings; excessive calcium may be harmful; and 20 weeks may be too late to alter outcomes.

**Objectives:**

To review the impact of lower dose calcium supplementation on pre-eclampsia risk.

**Search strategy and selection criteria:**

We searched PubMed and the Cochrane Pregnancy and Childbirth Group trials register.

**Data collection and analysis:**

Two authors extracted data from eligible randomised and quasi-randomised trials of low-dose calcium (LDC, <1 g/day), with or without other supplements.

**Main results:**

Pre-eclampsia was reduced consistently with LDC with or without co-supplements (nine trials, 2234 women, relative risk [RR] 0.38; 95% confidence interval [95% CI] 0.28–0.52), as well as for subgroups: LDC alone (four trials, 980 women, RR 0.36; 95% CI 0.23–0.57]); LDC plus linoleic acid (two trials, 134 women, RR 0.23; 95% CI 0.09–0.60); LDC plus vitamin D (two trials, 1060 women, RR 0.49; 0.31–0.78) and a trend for LDC plus antioxidants (one trial, 60 women, RR 0.24; 95% CI 0.06–1.01). Overall results were consistent with the single quality trial of LDC alone (171 women, RR 0.30; 95% CI 0.06–1.38). LDC plus antioxidants commencing at 8–12 weeks tended to reduce miscarriage (one trial, 60 women, RR 0.06; 95% CI 0.00–1.04).

**Conclusions:**

These limited data are consistent with LDC reducing the risk of pre-eclampsia; confirming this in sufficiently powered randomised controlled trials would have implications for current guidelines and their global implementation.

## Introduction

The hypertensive disorders of pregnancy cause maternal and perinatal death.^1^ Strategies to reduce the risk of hypertensive disorders of pregnancy are a global priority. Although pre-eclampsia is disproportionately prevalent in poor communities, an unexpectedly low prevalence was reported in Ethiopian and Guatemalan women with high levels of dietary calcium.^2^,^3^ Epidemiological, clinical and laboratory evidence suggests a role of dietary calcium deficiency in hypertensive disorders of pregnancy. In 2011 the World Health Organization recommended calcium supplementation with 1.5–2.0 g elemental calcium daily for pregnant women in areas with low dietary calcium.^4^ This recommendation was based on the evidence available from systematic reviews of randomised clinical trials, but raises several concerns.^4^

To date, systematic reviews have been limited to trials using high-dose calcium supplementation (1 g calcium/day or more), and excluded trials using smaller dosages. However, 1.5–2 g calcium/day exceeds the recommended daily allowance (RDA) of 1–1.3 g. Logistically, this dosage is heavy to transport: 1.5 g calcium-containing calcium carbonate plus glycine tablets weigh about 1 kg for a 20-week daily supply. For every 1000 pregnant women seen, 1000 kg of tablets is required. Ingesting three large tablets daily may be a barrier to compliance. Calcium is moderately expensive: the cost of chewable calcium carbonate tablets without vitamin D is $US 3–6/pregnancy (compared with $US 0.48 for iron supplementation).^5^ Calcium decreases iron absorption at doses of calcium >800 mg/day.^5^

Differences in dietary calcium intake between low-income and high-income countries approximate 500 mg. Typical daily intake in low-income countries ranges between 300 and 600 mg/day, compared with 855 mg (UK) and 969 mg (France).^6^

Excessive calcium supplementation may be harmful. The evidence for harm from excessive calcium is not conclusive. Calcium supplementation (but not dietary calcium) has been associated with myocardial infarction risk, but this Heidelberg study observation is at risk of confounding.^7^ Administration of 1.5 g calcium/day during pregnancy may cause rebound postnatal bone demineralisation (this Gambian finding is one of multiple comparisons and contrary to the a priori hypothesis)^8^ and we identified an unexpected increase in the syndrome of haemolysis, elevated liver enzymes and low platelets (HELLP) following calcium supplementation,^9^ perhaps through the antihypertensive effect of calcium masking the evolution of mild pre-eclampsia into HELLP syndrome (one of multiple comparisons).^10^

Although not conclusive, the possibility of adverse effects justifies efforts to determine the lowest effective dose of calcium supplementation (perhaps better considered to be calcium replacement up to the RDA) to reduce the effects of pre-eclampsia.

In view of the above concerns, we reviewed randomised trials of lower dosages of calcium supplementation in pregnancy (<1 g daily).

## Methods of the review

We searched the Cochrane Pregnancy and Childbirth Database for randomised trials of low-dose (<1 g/day) calcium supplementation during pregnancy, which included pre-eclampsia as an outcome, as well as searching PubMed for the terms ‘calcium AND (eclampsia OR pre-eclampsia OR hypertension) AND pregnancy AND (trial OR random)’.

We planned to include in the initial analysis, trials that met the following criteria: calcium supplementation without any co-supplements; random allocation with secure allocation concealment; and double blinding with placebo.

If the above criteria failed to produce adequate data, we planned to include data from quasi-randomised trials, trials without placebo control and trials of multiple supplements, with appropriate caution in the interpretation of such data.

We sought the following outcomes: pre-eclampsia; maternal death or serious morbidity; placental abruption; caesarean section; proteinuria; severe pre-eclampsia, as defined by trial authors; eclampsia; HELLP syndrome; intensive care unit admission; maternal death; maternal hospital admission ≥7 days; low birthweight (first weight obtained after birth <2500 g); neonate small-for-gestational age as defined by trial authors; neonate in intensive care unit for 7 days or more; death or severe neonatal morbidity; childhood disability; systolic blood pressure greater than 95th centile during childhood; and diastolic blood pressure greater than 95th centile during childhood.

Two authors assessed the risk of bias and extracted data from the original papers or translations of papers other than those in English using a purpose-designed data extraction form. We expressed outcomes as summary risk ratios with 95% confidence intervals (95% CI), using the Mantel–Haenszel method with a fixed effect model (RevMan software, Information Management System, Nordic Cochrane Centre, Copenhagen, Denmark). If there was significant heterogeneity we used a random effects model. Risk of bias was based on the adequacy of reported allocation concealment, and was categorised as: low risk of bias (e.g. telephone or central randomisation; consecutively numbered sealed opaque envelopes); or high risk of bias (open random allocation; unsealed or non-opaque envelopes, alternation; date of birth, or method not stated).

## Results

**Figure 1:**
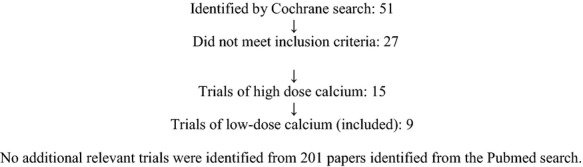
Flow diagram of study selection.

The PubMed search (17 September 2012) identified 201 papers, and the Cochrane Pregnancy and Childbirth Trials Register identified 51 trials, of which nine met the criteria for inclusion in this review (Figure1). One trial included a comparison that met our primary inclusion criteria (low risk of bias, low-dose calcium compared with no calcium).^11^ Both groups from this trial included in the review received low-dose aspirin as a co-intervention. Six trials did not report adequate allocation concealment strategies (high risk of bias), of which three used calcium alone^12^–^14^ and two used calcium plus Vitamin D.^15^,^16^ One trial with low risk of bias used calcium plus antioxidants^17^ and two trials with low risk of bias used calcium plus linoleic acid.^18^,^19^

**Figure 2:**
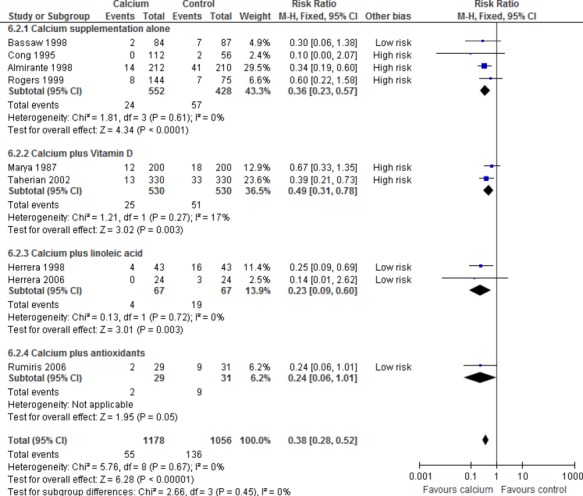
The effect of low-dose calcium supplementation in the second half of pregnancy with or without vitamin D, linoleic acid or antioxidants, on pre-eclampsia, including trials at low and high risk of bias.

The details of the included trials are shown in Table1. The effects of the interventions are shown in Table2 and Figure2. The four trials at low risk of bias, all in women at high risk of pre-eclampsia, showed a consistent reduction in pre-eclampsia (365 women, risk ratio 0.25; 95% CI 0.12–0.50). However, in three of these trials there was co-intervention with linoleic acid (two trials) or antioxidants.

**Table 1:** Features of the included studies

Study	Methods	Participants	Interventions included in this review	Risk of bias
Almirante (1998)^16^	‘divided into two groups and followed up until delivery’	430 nulliparous pregnant women who were adolescents and elderly (High risk of pre-eclampsia)	500 mg elemental calcium from 16 to 20 weeks till delivery versus controls	High
Bassaw (1998)^15^	Randomisation using a table of random numbers, supplements were distributed in sealed envelopes.	Pregnant women recruited before 20 weeks of gestation, primigravidae, or multigravidae with obstetric history of pre-eclampsia. No underlying medical disorders. (High risk of pre-eclampsia). Setting: Trinidad, women of African, East Indian and mixed ethnicity.	Included in this review: 600 mg elemental calcium plus 80 mg aspirin daily versus 80 mg aspirin (other groups studied were control and high-dose calcium alone).	Low
Cong (1995)^17^	‘randomised and divided into 3 groups’	Healthy primiparous women (Low risk of pre-eclampsia)	120 mg calcium daily versus 240 mg calcium daily (combined in this analysis) versus no calcium	High
Herrera (1998)^22^	Allocated to active tablets or identical-looking placebo by means of sequentially numbered, sealed allocation cards in computer-generated random sequence.	Primigravidas with risk factors for pre-eclampsia, positive roll-over test and high mean blood pressure; low dietary calcium (High risk of pre-eclampsia). Setting: Colombia, black and mixed race women, socio-economic levels 1 and 2.	450 mg linoleic acid plus 600 mg calcium versus placebo in the third trimester	Low
Herrera (2006)^23^	Allocated to active tablets or identical-looking placebo by means of sequentially numbered, sealed allocation cards in computer-generated random sequence.	Primigravidas <19 years or >35 years old, with risk factors for pre-eclampsia, abnormal uterine artery Doppler ultrasound, low dietary calcium (High risk of pre-eclampsia). Setting: Bangladesh and Colombia. Median daily dietary calcium 602 in the calcium group and 576 in the placebo group.	450 mg conjugated linoleic acid plus 600 mg calcium versus placebo from 18 to 22 weeks until delivery	Low
Marya (1987)^19^	‘Randomly selected’	Pregnant women 20–35 years old, low dietary calcium (Low risk of pre-eclampsia)	Calcium 375 mg plus Vit D 1200 IU from 20 to 24 weeks of pregnancy onwards versus control	High
Rogers (1999)^18^	Randomised in ratio 1:2:2 using five unsealed envelopes, selected by participants	Primiparous women in second trimester with rested left lateral automated blood pressureBP MAP 60 mmHg or more (Low risk of pre-eclampsia)	Calcium 600 mg daily from 22 to 32 weeks, then 1200 mg daily versus controls	High
Rumiris (2006)^21^	Double-blind, placebo-controlled trial. Randomised according to a computer-generated random number sequence by an independent third party.	Pregnant women with low antioxidant status at 8–12 weeks of gestation. No medical complications or current use of trial supplements. (High risk of pre-eclampsia). Setting, antenatal clinic, University of Indonesia.	Calcium 800 mg, *N*-acetylcysteine 200 mg, copper 2 mg, zinc 15 mg, manganese 0.5 mg, and selenium 100 μg and vitamins A 1000 IU, B6 2.2 mg, B12 2.2 μg, C 200 mg and E 400 IU, from 8 to 12 weeks of gestation throughout pregnancy.	Low
Taherian (2002)^20^	‘randomised and divided into 3 groups’	Healthy nulliparous women (Low risk of pre-eclampsia)	500 mg calcium + 200 IU vitamin D from 20th week of pregnancy till delivery versus control	High

**Table 2:** Results of meta-analysis of trials with low risk of bias

Outcome or subgroup	Studies	Participants	Effect estimates*
**High blood pressure**	2	219	0.42 (0.20–0.87)
Calcium supplementation alone	1	171	0.60 (0.25–1.46)
Calcium plus linoleic acid	1	48	0.20 (0.05–0.82)
**Pre-eclampsia**	4	365	0.25 (0.12–0.50)
Calcium supplementation alone	1	171	0.30 (0.06–1.30)
Calcium plus linoleic acid	2	134	0.23 (0.09–0.60)
Calcium plus antioxidants	1	60	0.24 (0.06–1.01)
**Caesarean section**	2	134	0.55 (0.34–0.86)
Calcium plus linoleic acid	2	134	0.55 (0.34–0.86)
**Severe pre-eclampsia**	2	146	0.34 (0.10–1.21)
Calcium plus linoleic acid	1	86	0.33 (0.07–1.56)
Calcium plus antioxidants	1	60	0.36 (0.04–3.23)
**Preterm birth**	2	108	0.41 (0.08–2.05)
Calcium plus linoleic acid	1	48	0.50 (0.05–5.15)
Calcium plus antioxidants	1	60	0.36 (0.04–3.23)
**Birthweight <2500 g**	2	134	0.20 (0.05–0.88)
Calcium plus linoleic acid	2	134	0.20 (0.05–0.88)
**Neonate small for gestational age**	3	194	0.38 (0.10–1.38)
Calcium plus linoleic acid	2	134	0.29 (0.06–1.32)
Calcium plus antioxidants	1	60	1.07 (0.07–16.31)
**Stillbirth or death before discharge**	4	365	0.61 (0.15–2.53)
Calcium supplementation alone	1	171	1.04 (0.07–16.29)
Calcium plus linoleic acid	2	134	0.60 (0.08–4.41)
Calcium plus antioxidants	1	60	0.36 (0.02–8.39)
**Miscarriage (not prespecified)**	1	60	0.06 (0.00–1.04)
Calcium plus antioxidants			0.06 (0.00–1.04)

Low-dose calcium supplementation (<1 g/day) with or without co-supplements.

* Effect estimates expressed as risk ratio (95% CI), Mantel–Haenszel method, fixed effects model.

## Discussion

### Main findings

Trials of low-dose calcium supplementation in women at high risk of pre-eclampsia, without or with linoleic acid or antioxidants, report a reduction in pre-eclampsia similar to that shown in the trials of high-dose calcium supplementation in women at high risk of pre-eclampsia. Whether the effect in the trial with antioxidants can be attributed in part to the antioxidants is unlikely, as a systematic review found that antioxidants do not reduce the risk of pre-eclampsia.^20^ We are not aware of robust evidence regarding possible effects of linoleic acid on pre-eclampsia.

### Limitations

Due to the limitations of the studies included (which included trials with concurrent antioxidant administration), the findings of this review should not be regarded as conclusive. However, they are consistent with the possibility of a beneficial effect of low-dose calcium supplementation during pregnancy, and, therefore, highlight the need for methodologically sound, sufficiently powered trials to either confirm or refute this effect.

### Interpretation

The findings of this review, although studying calcium supplementation in late pregnancy, rather than before pregnancy and in early pregnancy, support our principle of studying lower-dose calcium supplementation in relation to the hypertensive disorders of pregnancy. Currently, we are conducting a trial of low-dose calcium supplementation (500 mg/day) in women with previous pre-eclampsia, commencing before pregnancy and continuing for up to 20 weeks of pregnancy.^8^ The choice of the dose of calcium was based on practical considerations: if calcium supplementation before and in early pregnancy is found to have an important effect on the development of pre-eclampsia, the only viable way of implementing the intervention at a community level would be with food fortification. The dosage of 500 mg would be achievable with fortification of calcium-poor staple foods, without exposing the general population to levels of dietary calcium in excess of the RDA. Food calcium fortification is seen as a relevant strategy not only for prevention of pre-eclampsia but also for bone health and for lowering the risk of other disorders such as hypertension, colon cancer and obesity.^10^

The RDA for calcium intake during pregnancy has been a subject of controversy. Based on considerations of fetal needs and high calcium urinary output in pregnancy, historical figures for calcium intake as high as 2 g/day have been proposed to obtain a positive balance during pregnancy.^21^ However, it has been reported that calcium absorption in pregnancy reaches levels twice as high as those seen in nonpregnant women.^22^ The current National Institutes of Health (NIH) RDA for pregnant women aged 14–18 years is 1300 mg, and 1000 mg for pregnant women aged 19–50 years.^23^ Health Canada RDA replicates NIH, and The Singapore Health Promotion Board RDA for calcium during pregnancy is 1000 mg. Reduced dietary calcium intake below the RDA is associated with hypertension outside pregnancy,^24^ as well as osteoporosis, renal stones, increased body mass index, insulin resistance and colorectal cancer.

It should be recognised that the epidemiological association of pre-eclampsia with low dietary calcium is not evidence of a direct causal link. Efforts to confirm such a link have focused on randomised trials of calcium supplementation during pregnancy, commencing in the late 1980s.

Previously, we conducted a systematic review of randomised trials of calcium supplementation of at least 1 g daily during pregnancy.^9^ This review discussed the epidemiological, clinical and laboratory studies linking pre-eclampsia with dietary calcium deficiency. The results of the review showed inconsistency between the trials in women at high and low risk of pre-eclampsia. The risk ratio (RR) for pre-eclampsia for trials in women at high risk of pre-eclampsia was 0.22 (95% CI 0.12–0.42). The risk ratio for trials in women at low risk was 0.59 (95% CI 0.41–0.83). The results of the review were dominated by the WHO trial of calcium supplementation among low-calcium-intake pregnant women conducted in 2001–03.^10^ Results from this trial showed that although 1.5 g calcium/day in the second half of pregnancy did not prevent pre-eclampsia (RR 0.92, 95% CI 0.75–1.13), there was a significant reduction in the outcome maternal death or severe morbidity (RR 0.80, 95% CI 0.65–0.97), including a trend to fewer deaths with calcium supplementation (1/4151 versus 6/4161 women).

Other reviews have concluded that calcium supplementation is an effective intervention to reduce pre-eclampsia,^11^ neonatal mortality and preterm birth in developing countries.^25^

In addition, we have reported that high-dose calcium supplementation in pregnancy reduces the serious adverse effects of pre-eclampsia, but has no effect on markers for pre-eclampsia (proteinuria, platelet counts or urate levels).^7^ We proposed the hypothesis that calcium supplementation in the second half of pregnancy might reduce blood pressure and, therefore, the serious complications of this component of pre-eclampsia without affecting other components of the syndrome or the underlying pathology related to placental development, and that calcium supplementation before and in early pregnancy might affect this process.^25^

Indirect evidence that supra-physiological doses of calcium may not improve outcomes above physiological dosages is given by the large CPEP study (4589 women), which compared calcium 2 g daily versus placebo in women with normal calcium intake. Actual median intakes were 2369 g in the calcium group versus 982 g with placebo. This additional supplementation of women with normal dietary calcium had no statistically significant effect on pre-eclampsia. In addition, we have outlined the caveats around high-dose calcium supplementation related to the Heidelberg and Gambian studies, above.^7^,^8^

## Conclusions

We have not identified robust evidence related to the effect of low-dose calcium supplementation in pregnancy on pre-eclampsia and related outcomes. The available evidence is consistent with a reduction in the risk of pre-eclampsia. Confirmation of this possible effect in sufficiently powered, robust randomised trials would have major implications for the current WHO guidelines and their implementation in low-income countries.

Currently, the logistics and cost of the WHO recommendation for supplementation with 1.5–2 g calcium daily have been regarded as prohibitive in several settings (Smith JM. [Internet]. E-mail to Diane Sawchuck. 2012 December 12 [cited 2013 Feb 12]). The dilemma facing health policymakers in these settings is whether supplementation with a lower dose would be better than no supplementation at all. The findings of this review and commentary provide limited evidence on which to base such decisions, but further research is needed.

### Disclosure of of interest

None to declare.

### Contribution to authorship

GJH and JMB conceived the review, did the initial analysis and wrote the first draft of the pages. PVD substantially revised the first draft. GJH, JMB and PVD contributed to the literature search, data analysis, data interpretation and/or the writing of the report.

### Funding

This work was supported by a grant to the Effective Care Research Unit from the University of British Columbia, a grantee of the Bill & Melinda Gates Foundation. The sponsors of the study had no role in study design, data collection, data analysis or interpretation but did review this report before submission for publication. The corresponding author had full access to all the data in the study and had final responsibility for the decision to submit for publication.
